# The Effects of a Multicomponent Social Support Intervention on Physical Fitness and Exercise Attitude in Children: A 12-Week Randomized Controlled Trial

**DOI:** 10.3390/ijerph19169922

**Published:** 2022-08-11

**Authors:** Yijuan Lu, Kehong Yu, Jun Jin, Xiaomei Gan

**Affiliations:** 1 Department of Sport Science, College of Education, Zhejiang University, Hangzhou 310027, China; 2 Center for Sports Modernization and Development, Zhejiang University, Hangzhou 310027, China; 3 Wensan Education Group, Wensan Street Primary School, Hangzhou 310061, China

**Keywords:** students, RCT, instrumental social support, physical health, promotion

## Abstract

Purpose: The current study is intended to assess the effects of a multicomponent social support intervention program on grip strength, sprinting, jumping, agility, flexibility, core strength and exercise attitude among children over a period of twelve weeks. Method: This study followed a randomized parallel design in which 366 children (age: 9.35 ± 1.12 years) in the 3rd–5th grade at an elementary school in China were allocated to an intervention group (*n* = 183) or a control group (*n* = 183). Participants were assessed twice (baseline and postintervention) for the following tests: (i) grip strength, (ii) 50 m dash, (iii) rope skipping, (iv) sit-ups, (v) sit-and-reach, and (vi) exercise attitude. The intervention group received the “Exercise Methods and Wellness Knowledge Guide (EMWKG)” leaflet three times a week. The control group was not required to make any changes. Results: Significant increases in pre–post left-hand grip strength (Cohen’s d = 0.10, *p* < 0.001), right-hand grip strength (Cohen’s d = 0.09, *p* < 0.001), and behavioral intention (Cohen’s d = 0.17, *p* < 0.01) were observed in the intervention group. Students in the control group showed significant decreases in three indicators: both 50 m (Cohen’s d = 0.19, *p* < 0.01) and sit-ups (Cohen’s d = 0.14, *p* < 0.05) as well as sit-and-reach (Cohen’s d = 0.46, *p* < 0.001). Significant differences (*p* < 0.001) were found between the two groups in right-hand grip strength (*F* = 7.109, *η_p_*^2^ = 0.020), sit-and-reach (*F* = 11.255, *η_p_*^2^ = 0.031), and subjective standards (*F* = 15.461, *η_p_*^2^ = 0.043). A comparison of the post hoc test results showed that the intervention group scored 0.519 kg (95% CI: 0.136–0.901, Cohen’s d = 0.27), 0.944 cm (95% CI: 0.0391–1.497, Cohen’s d = 0.35), and 2.535 points (95% CI: 1.267–3.803, Cohen’s d = 0.41) higher than the control group in right-hand grip strength, sit-and-reach, and subjective standards, respectively. Conclusion: This combined social support theory-based intervention was effective in improving exercise attitude and fitness among children in 12 weeks.

## 1. Introduction

The decline in the physical health of children as a result of sedentary lifestyles is a public health problem affecting more than half of the world’s adolescent population in both developed and developing countries [1]. The level of physical activity in childhood is not only closely related to children’s physical and mental health but also has an impact on their exercise habits in adulthood [2,3]. According to survey results, Chinese children participate in more hours of moderate-to-vigorous physical activity in-school [4,5], while foreign children tend to participate in more moderate-to-vigorous physical activity outside school [5,6,7,8].

Intervention studies have placed a particular emphasis on the role of the family, and studies have demonstrated the positive effects of family-level interventions on long-term changes in the physical activity of students [9,10], as well as the positive effects of including a family component in school interventions [11]. Education is the most common intervention in family-level interventions, and in one study included in a systematic review by Brown [12], 80% of the interventions included an educational component. The effectiveness of education in improving students’ knowledge concerning physical activity has been demonstrated. Chen et al. [13] and Beech et al. [14] showed that education is effective in changing physical activity knowledge. Pittman [15] used peer social networking sites and text messaging to intervene in children’s BMI, body fat percentage, physical fitness, and physical activity self-efficacy. Noar et al., conducted a meta-analysis of studies on the effectiveness of targeted, printed materials to intervene in health behaviors and found that targeted information interventions had significant impacts on the health behaviors of subjects in an experimental group in contrast with a control group, and that colorful printed materials were more effective than message interventions for healthy behaviors [16]. While affirming the positive intervention effects of education, some researchers have suggested that capturing readers’ attention during information-based interventions is a key prerequisite for the effectiveness of health education messages [17], that education alone (e.g., providing email-based information about physical activity) is not sufficient to change students’ behavior, and that interventions without additional strategies may be ineffective. In their study, Arredondo et al. [18] highlighted two factors that are of concern, namely, the lack of parental knowledge and access to resources.

In response to these two factors, researchers have affirmed the role of social support, in particular the importance of parental support. The study of social support theory in psychology began in the 1960s in the context of the exploration of the effects of life stress on physical and mental health. However, it was not until the 1970s that social support was first introduced as a professional concept in the psychiatric literature by Cassel (1976) [19]. Since then, it has been widely and intensively discussed and studied as an important issue by many prominent researchers. Intervention studies [20] and longitudinal studies [21] have shown that after social support interventions, in addition to these interventions causing students to engage in more physical activity, students who received more social support experienced smaller decreases in physical activity levels over time. In a systematic review addressing social support and physical activity in adolescents, Mendonc et al. [22] also noted that social support not only one of the important factors for adolescents’ participation in physical activity but also is an important factor in their continued participation in exercise and that it should be included in intervention programs to increase their physical activity levels and to promote physical fitness and health development. A longitudinal study by Davison and Jago [23] showed that the influence of social support on physical activity was directly related to parental influence in the early stages, while the influence of friends on physical activity increased in late adolescence. The same variability between the two was found after studying social support in different activity domains and from different sources. Mendonca et al. [22] found that parental support as well as friend social support were positively associated with physical activity in spare time. However, in commuting physical activities, only friend support showed a positive association. A follow-up study found that the effect of the intervention differed between different types of support from the same source of social support. In a systematic review of parental support, Rhodes et al. [24] found that the types of parental support included providing information, attending and watching, home environment facilities, joint participation, supervision, encouragement, and logistical support, with parental encouragement, logistical support, and joint participation being more common in intervention studies.

In China, since 1 January 2022, the *Law on the Promotion of Family Education* has been officially implemented. It is clear that parents or other guardians should establish a sense of responsibility of the family being the first classroom and that parents are the first teachers, assuming the primary responsibility for implementing family education for minors; educating minors on correct concepts; and developing methods and behaviors for effective thinking, behavior, and habits. Teaching by example is more important than teaching by word, but studies have found that parents seem to be overly dependent on school education, and they are often absent in their own roles in guiding and motivating their children’s physical activity behaviors. Children who lack the ability to make their own judgments are precisely the most susceptible to parental influence, and parental guidance and participation are both a priority and a challenge for children’s physical activity practices [25]. In order to promote children’s physical activity, Chinese education authorities have proposed the requirement of one hour in school and one hour out of school for physical exercise. The necessity of physical education homework was affirmed in the *Notice on Further Strengthening the Management of Physical Fitness of Middle School Children* issued by the Chinese Ministry of Education, which emphasizes efforts to guarantee students one hour of physical activity each day in and out of school and specifies a physical education homework system. In the *Physical Education and Health Curriculum Standards* (2022 Edition) [26], it is required that students are physical education homework that is to be completed independently or cooperatively, and jointly with parents. Duncan et al. [27] argue that physical education homework aimed at family participation can promote family engagement, improved family relationships, and even healthier lifestyles for the whole family. Especially in the context of the normalization of epidemic preparedness, a child’s home time is prolonged, and the importance of family education is high.

It follows that securing family physical education support to promote children’s exercise perceptions, habits, and behaviors is the focus of interventions. However, most of the research on physical fitness promotion in China is focused on school-based interventions, with 40% of the studies using school recess as a vehicle for intervention. Chinese parents seem to be overly dependent on school education, their role in guiding and motivating their children’s physical activity behaviors is often absent, and their knowledge base and practical skills related to physical activity behaviors are insufficient. Thus, our study is based on social support theory and proposes intervention elements based on four categories: emotional support, substantive support, informational support, and evaluation support. Based on this concept, we created an “Exercise Methods and Wellness Knowledge Guide”(EMWKG) for parents and students to learn and practice together, in accordance with the physical education homework assigned by the pilot school.

Based on the above, this study (1) created the EMWKG for children in grades 3–5; (2) designed the intervention program based on social support theory; and (3) aimed to evaluate the effect of the intervention program on improving children’s attitudes toward exercise and physical performance.

## 2. Materials and Methods

### 2.1. Study Design

**Figure 1:**
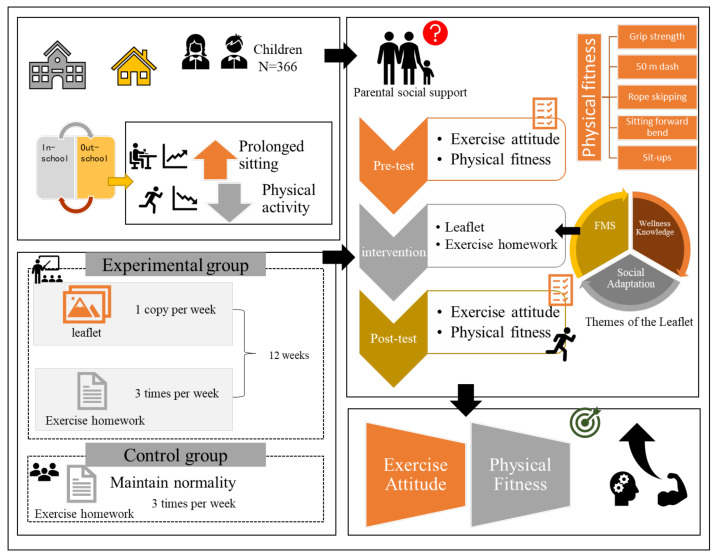
Research design flow chat.

A 12-week randomized controlled trial (RCT) was conducted between September 2020 and January 2021 and used a single-blind design (as shown in Figure 1). For subject recruitment, the researchers contacted the experimental school principal, class adviser, and physical education teacher in advance. The researcher entered the class during the class meeting and used a PPT format to deliver the experiment. Informed consent forms (signed by both parents and students) were distributed after the presentation. The signed informed consent form was collected by the classroom teacher the next day. Participants were randomly assigned to one of two groups prior to the commencement of the study using a simple computer-generated randomization process [28]. The active intervention lasted 12 weeks in this 15-week trial, while recruitment, screening, pre-measurement, and post-measurement took up the remaining 3 weeks. More specifically, the first week was spent screening and recruiting potential subjects. The following week, all students completed questionnaires and physical fitness tests. Finally, in week 15, a post-test procedure similar to the baseline procedure was completed after the 12-week period.

Our study proposed corresponding intervention elements based on four classifications of social support theory (emotional social support, instrumental social support, informational social support, and evaluative social support) and designed intervention programs according to the proposed elements (shown in Table 1).

Based on the intervention elements, we developed the EMWKG around three themes: physical health knowledge, fundamental movement skills, and social adaptation to sport. The guide was for parents and students to learn and practice together. The EMWKG flipchart content (shown in Table 2) was prepared differently for third and fifth grade, and the fifth-grade content was an enhancement of the third-grade content. Because a child’s physical and mental development is stage-specific, there are significant differences in physical and mental development between students in lower grades and those in upper grades.

### 2.2. Participants

**Figure 2:**
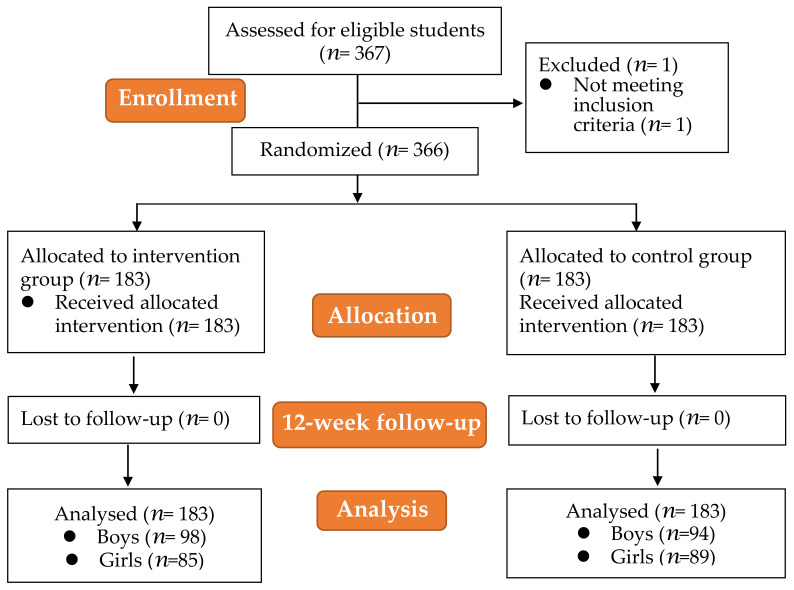
Flow diagram of the study participant selection.

A random sample of 367 students from 8 classes in an elementary school in Hangzhou, Zhejiang Province, China, who voluntarily participated in this study were selected. Because children in grades 1 and 2 were cognitively limited and unable to complete the questionnaire independently, this study was conducted on a random sample of grades 3–5 only. The enrolled students were required to be able perform physical fitness tests (e.g., sprints, sit-ups, rope skipping) and not participate in the school’s after-school sports club. A total of 367 candidates were recruited for the study, and 366 volunteers met the inclusion criteria; one was excluded due to a medical condition that prevented him from participating and completing the physical fitness test, as described in Figure 2. Prior to the study, we collected demographic information on these students and then assigned them to the intervention group (*n* = 183) or the control group (*n* = 183). A total of 366 students received and completed a baseline test (containing a psychological questionnaire test and physical fitness tests), a 12-week follow-up examination, and a second repeat test.

As a result, data from questionnaires and physical fitness tests were screened and cleaned for a total of 183 students (98 males and 85 females) in the intervention group and 183 participants (94 males and 89 females) in the control group (shown in Table 3). The students’ mean age was 9.35 ± 1.12 years. All pupils and families were thoroughly informed about any potential issues with the experimental procedures. The Zhejiang University Research Ethics Committee authorized the study protocols (No. 2020-015, 1 August 2020). All participants provided written informed consent.

### 2.3. Intervention

**Figure 3:**
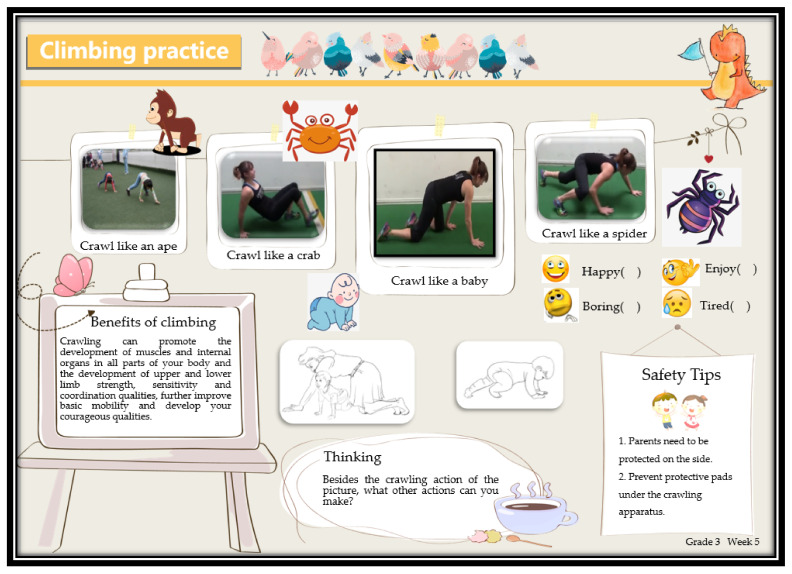
Contents of the third-grade week 5 flipchart. Crawling practice topics (introduction to the benefits of crawling, demonstration of various forms of crawling practice, safety and protection knowledge).

**Figure 4:**
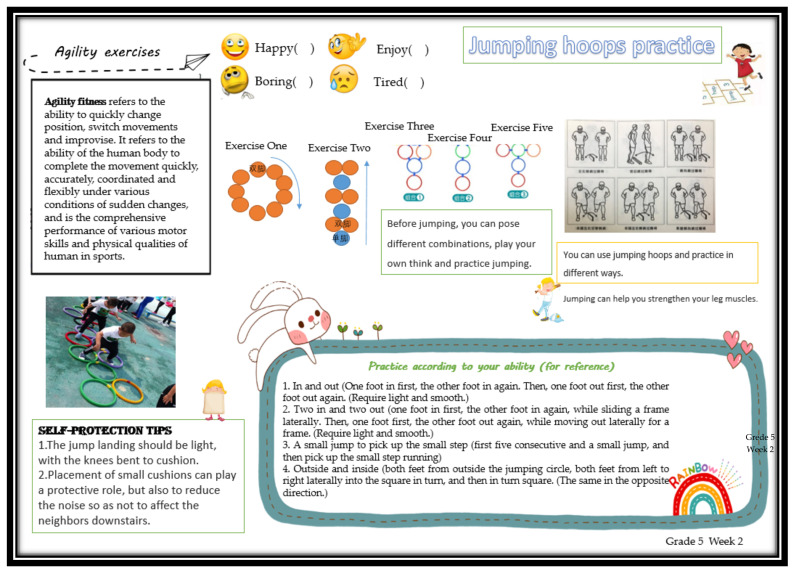
Contents of the flipchart for the second week of the fifth grade. Sensitivity practice topics (introduction to what is meant by sensitivity, demonstration of various forms of sensitivity practice, knowledge of safety and protection).

The experimental group completed the “Exercise Methods and Wellness Knowledge Guide” (EMWKG) leaflet intervention in addition to the original PE homework. The EMWKG flipchart is based on several educational themes, using a combination of text and cartoon illustrations to stimulate children’s interest in learning including learning knowledge and skills outside the classroom to enhance the health of the children and their families. For example, Figure 3 and Figure 4, respectively, present the contents of the flipchart for the fifth week of the third grade and the second week of the fifth grade. One page of loose-leaf printed intervention materials was distributed weekly to the test group students by the physical education teacher, and the intervention materials were provided one week in advance by the investigator. The total intervention period was 12 weeks, with materials distributed every Monday. The themes of each week’s intervention are shown in Table 4. At the first distribution, each student was provided with a cartoon plastic file bag to hold the weekly intervention materials and a free set of agility ladder practice equipment. Each printed page had four cartoon options for students to check off in response to the content of the booklet: happy, like, bored, and very tired. students’ booklets were collected by the researcher after 12 weeks of intervention. Students in the intervention group were told that they could not share EMWKG materials with their classmates.

The control group only completed the physical education homework and did not perform the EMWKG loose-leaf intervention or receive the agility ladder exercise equipment.

### 2.4. Measurements

#### 2.4.1. Exercise Attitude Test

The Exercise Attitude Scale [29] created by Mao was used in this study to gauge the attitudes of the participants toward exercise. Details of this test are described in Lu et al. [30]. The researchers collected data on the exercise attitude of the third graders, which was different from that of the fourth to sixth graders. In order to help the third-grade subjects better understand and fill out the psychological questionnaire, the researcher read each question one by one, and the students answered each question until they completed the entire content. After the students had completed the questionnaire, the researcher asked the students if they had any questions about the content of the questionnaire or whether they needed any help.

#### 2.4.2. Physical Fitness Test

The tests were arranged in the order of grip strength, 50 m dash, sit-ups, rope skipping, and sit-and-reach. The procedure for the grip strength and 50 m tests can be found in Lu et al., (2022) [30]. For the sit-ups test, the subject lies on his or her back on a mat with legs slightly apart, knees bent at an angle of approximately 90 degrees, and two fingers crossed against the back of the head. A partner presses the ankle joint to fix the lower limbs. The subject sits up with both elbows touching or extending past both knees once. Both scapulae must touch the mat when lying supine. When the test person gives the “start” command, he or she opens the table and records the number of sit-ups completed within 1 min. One minute was not counted if the subject sat up but the elbows did not reach both knees. If it was found that the subject used the elbow pad or the rise and fall of the hip to sit up, that time was not counted. During the test, the observer reports the number to the subject. Before the rope-skipping test, subjects adjusted the length of the rope to the appropriate length and stood with their legs together in a natural position. The test was measured using an electronic jump rope, and when the signal to start the test was heard, the subjects commenced the test. At the end of the test, the researcher recorded the test values. If the subject jumped on one foot or alternately, the number of jumps was not counted. The tester was to correct immediately and then to continue the test. Before the sit-and-reach test, the subject was to prepare for the activity on a flat surface to prevent strain. The subject sat on a soft cushion attached to a box, both legs straight, not bent, with heels together and toes apart about 10–15 cm, stepping on the flat vertical plate of the measuring meter and both hands together. When testing, with two arms and hands straight, the subjects gradually brought the upper body into forward flexion with the fingertips of the two hands gently pushing the scale on the vernier forward slide (there was not to be sudden forward movement) until they could not continue to reach forward. The test meter’s foot stirrup longitudinal plate inside the plane was the 0 point, inward for a negative value and forward for a positive value. The value was recorded in centimeters, using one decimal point. If the value was positive, then a + symbol was added before the value, and if negative, a − symbol was added.

### 2.5. Statistical Procedures

IBM SPSS statistics 25 and GraphPad software were used to calculate the descriptive statistics, *t*-tests, and analysis of covariance (ANCOVA) on the data. We used analysis of covariance to determine the effects of the intervention using baseline scores and other key confounders (age, BMI) as covariates and experimental condition and gender as fixed variables. Traditionally, intervention effects are tested using repeated-measures ANOVA, which emphasizes that subjects do not differ significantly at the baseline level. In this study exploring the effect of a certain intervention method (experimental variable) on students’ physical fitness test scores (experimental effect), the subjects’ preexisting baseline physical fitness affected their physical fitness test scores, but it is often difficult to select subjects with the same physical fitness baseline to participate in an experiment; for this reason, this study used baseline scores as covariates to improve statistical power and precision [31]. After confirming the significant interaction of overall ANCOVA, simple-effects analysis and post hoc Bonferroni adjustment were performed. The statistical significance of all tests was set at *p* < 0.05. The presence of an interaction between gender and intervention modality was judged by the *p*-value of the F-test (probability of the null hypothesis being true in the F-test) at 0.05. If p is greater than 0.05, it means that the interaction term is not statistically significant and there is no interaction between gender and intervention mode; if p is less than or equal to 0.05, it means that the interaction term is statistically significant and there is an interaction between gender and intervention mode. The effect of the intervention modality was determined using main effects analysis based on the difference in means, Cohen’s d, and corresponding *p*-values for the different groups when there was no interaction. When there was an interaction, main effects were analyzed to determine the superiority of the intervention according to sex based on the mean difference and the corresponding *p*-value between females and males under different intervention modalities; alternatively, interaction control analysis was used to analyze the sex difference in the same group using comparisons (post hoc) between multiple groups, specifying which groups differed in the mean difference.

Effects sizes were calculated as *η*^2^*_p_* and graded as follows: small, 0.01 ≤ *η*^2^*_p_* < 0.06; medium, 0.06 ≤ *η*^2^*_p_* < 0.14; or large, *η*^2^*_p_* ≤ 0.14 [32]. The statistical significance of all our tests was set at *p* < 0.05. Effect sizes for differences in means were expressed as Cohen’s d (difference in means divided by the standard deviation of the difference), with 0.2, 0.5, and 0.8 indicating small, medium, and large effect sizes, respectively [33].

## 3. Results

### 3.1. Differences between Children in the Intervention and Control Groups at Baseline

The results for the differences between groups at baseline testing showed no significant differences in the components of the fitness test and the exercise attitude test, except for significant differences in behavioral attitudes and behavioral cognition (*p* > 0.05). The chi-square of the gender difference between the two groups was 0.175 (*p* ≥ 0.05).

### 3.2. Effects of Intervention on Childrens’ Physical Fitness

**Figure 5:**
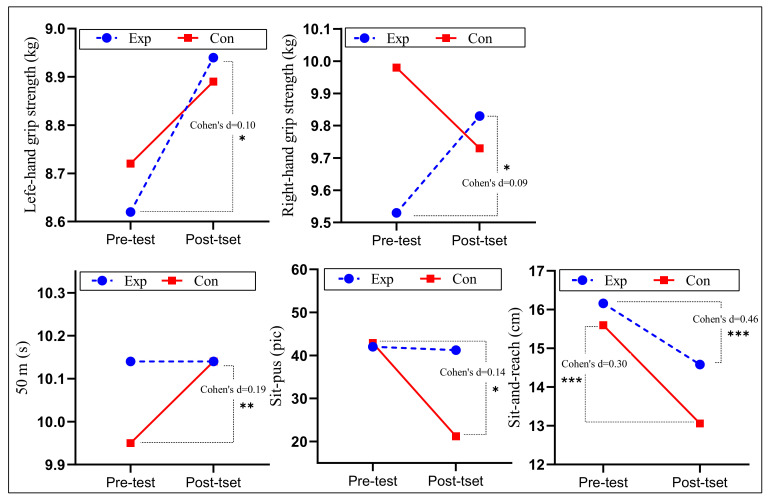
Descriptive statistics and within-group variations in the means of physical fitness. *: *p* < 0.05; **: *p* < 0.01; ***: *p* < 0.001.

The results for the intra-group differences before and after the intervention in the control group are shown (Table 5 and Figure 5). The intervention group showed significant improvements in both left–and right–handed grip-strength tests (Cohen’s d = 0.10 and 0.09, *p* < 0.05), but a significant decrease in sit-and-reach (Cohen’s d 0.3, *p* < 0.001). Students in the control group showed significant decreases in three indicators: 50 m (Cohen’s d = 0.19, *p* < 0.01), sit-ups (Cohen’s d = 0.14, *p* < 0.05), as well as sit-and-reach (Cohen’s d = 0.46, *p* < 0.001).

**Figure 6:**
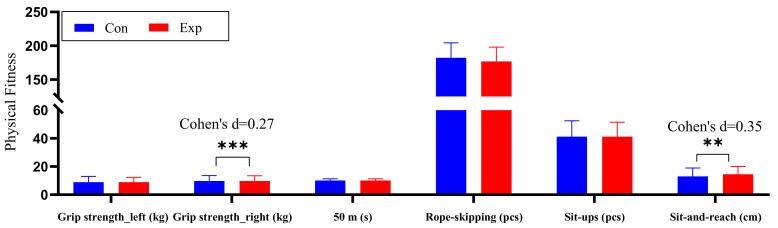
Descriptive statistics, between-group variations of means of physical fitness. **: *p* < 0.01; ***: *p* < 0.001.

Rapid physical and intellectual development during childhood is characterized by significant gender differences. For this reason, we include gender as a factor. After controlling for age, BMI and baseline scores, the results of the ANOVA showed significant group effects for right-hand grip strength (*F* = 7.109, *p* < 0.001, *η*^2^*_p_* = 0.020) and sit-and-reach (*F* = 11.255, *p* < 0.001, *η*^2^*_p_* = 0.031) in the physical performance component. We found no significant interactions between groups or for gender in the mixed ANCOVA for grip strength (*p* = 0.150/0.150, *η*^2^*_p_* = 0.006/0.006), 50-m dash (*p* = 0.876, *η*^2^*_p_* < 0.001), rope skipping (*p* = 0.991, *η*^2^*_p_* < 0.001), sit-ups (*p* = 0.280, *η*^2^*_p_* = 0.003), or sit-and-reach (*p* = 0.071, *η*^2^*_p_* = 0.009). Table 6 and Figure 6 present the results from between-group analyses.

Bonferroni post hoc comparisons showed that in terms of the effect on right-hand grip strength, the experimental group gripped 0.519 kg (95% CI: 0.136–0.901, Cohen’s d = 0.27) more than the control group. In the effect on sit-and-reach, students in the experimental group reached 0.944 cm (95% CI: 0.0391–1.497, Cohen’s d = 0.35) farther than those in the control group.

### 3.3. Effects of Intervention on Childrens’ Exercise Attitudes

**Figure 7:**
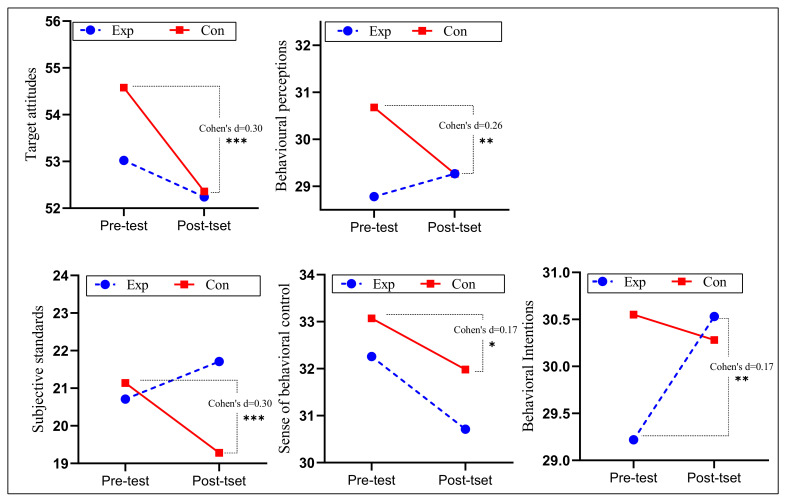
Descriptive statistics and within-group variations of means of exercise attitude. *: *p* < 0.05; **: *p* < 0.01; ***: *p* < 0.001.

Table 7 and Figure 7 give the descriptive statistics for the students’ within-group preintervention and postintervention attitudes toward exercise. Students in the experimental group showed improved behavioral perceptions, behavioral habits, emotional experience, subjective standards, and behavioral intentions in the exercise attitude component compared with pre-intervention, with behavioral intention (Cohen’s d = 0.17, *p* < 0.01) being significantly higher. The control group showed significantly lower target attitudes (Cohen’s d = 0.3, *p* < 0.001), behavioral perceptions (Cohen’s d = 0.26, *p* < 0.01), sense of behavioral control (Cohen’s d = 0.17, *p* < 0.05), and subjective standards (Cohen’s d = 0.30, *p* < 0.001) in the exercise attitude component compared with the baseline test.

**Figure 8:**
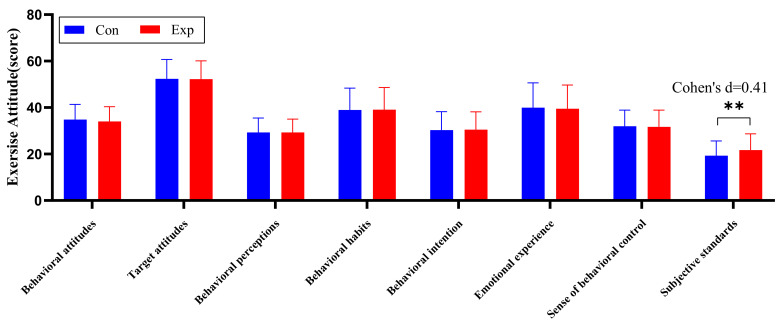
Descriptive statistics and between-group variations of means of exercise attitude. **: *p* < 0.01.

After adjusting for covariates, we found non-significant differences in the exercise attitudes component, except for subjective standards (*F* = 15.461, *p* < 0.001, *η*^2^*_p_* = 0.043), which showed statistically significant differences between groups (Shown in Table 8). We also found no significant interaction effects for behavioral attitudes, target attitudes, behavioral perceptions, behavioral habits, behavioral intentions, emotional experiences, sense of behavioral control, or subjective standards. Bonferroni post hoc comparison showed that the subjective standards score was 2.535 points (95% CI: 1.267–3.803, *p* < 0.05, Cohen’s d = 0.41) higher in the experimental group than in the control group (Shown in Figure 8).

## 4. Discussion

In order to make the experimental design as rigorous and scientifically sound as possible, the control and experimental classes of students were controlled for variables related to the physical education curriculum, extracurricular physical activity, and physical education homework that might have had an impact on the experimental results in addition to that of the intervention of the EMWKG flipchart. The intervention content of the study design was developed separately for the specific target population (third or fifth grade). The intervention was conducted once a week with an EMWKG loose-leaf intervention to allow the students to learn the exercise methods and wellness knowledge each week as much as possible, with constant reinforcement, and to allow students to review what they had learned the previous week. Our study combined the characteristics of children’s physical and mental development at different stages, their needs, the laws of motor development, and the sequence of physical ability development to design targeted EMWKG flipcharts for groups of children. The study also presented physical education knowledge and exercise content and methods in the form of cartoon drawings to enhance students’ interest. The EMWKG flipchart was designed not only to include the motor skills section, but also to introduce wellness knowledge, such as that pertaining to exercise and obesity, exercise and nutrition, and exercise and eye health, so that students can understand the benefits and importance of exercise for their bodies, thus improving their attitudes and changing their physical activity behaviors.

According to the theory of planned behavior and the theory of perceived behavioral control [34], both attitudes and subjective standards are important determinants of a person’s intention to engage in a particular behavior [35]. In influence on behavior, behavioral intention is the most proximal determinant, and behavioral intention is determined by attitudes and subjective standards. After the experimental group read and studied the flipchart with their parents, their motivation for physical exercise increased and their satisfaction in performing physical exercise improved. It not only caused the numbers and times of exercise to increase, but the exercises were more scientific in method and were effective under the guidance of the flipchart. As a result, this caused the students in the experimental group to show more obvious progress in strength quality and speed quality.

Our study is based on the interpersonal system of the socioecological model, with an integrated intervention using the home-school as a vehicle, taking into account parental influences and targeting both parental support and teacher support factors of the interpersonal ecological subsystem. The transmission of social support affects the effectiveness of the intervention [18]. Classroom teachers have a particular influence on children’s behavior [36] and spend the most time with students at school, where interventions are more effective through a classroom teacher’s words (e.g., “you need to read carefully and actively follow the content”) and behaviors (e.g., the weekly distribution of colorful flipcharts) than in person by the researcher. For this reason, the teacher support intervention in the school setting was designed so that the distribution of the weekly one-page colorful flipchart was a task for the classroom teacher in each classroom. The design of the parental support interventions in the family environment also included cartoon illustrations of exercises with parents, which not only enabled parents to learn about physical health and physical exercise and to be able to guide their children’s exercise more scientifically but also increased the time for joint exercise involving parents and children. According to research, many parents are willing to help their children to exercise, but half of them do not actually do it, emphasizing the benefits of physical activity rather than acting together, leading to an “intention-behavior gap” [12,24,37,38,39]. Therefore, an effective intervention is one that equips parents with the knowledge and skills for how to exercise, rather than why to do so [12]. According to the students’ questionnaire responses, there were significant differences in the students’ subjective ratings after the intervention. The scores of the items “My parents often tell me to exercise”, “I am more influenced by my parents about exercise”, and “I take my parents’ advice whether to exercise or not” increased. This shows that a good, family sporting environment has a positive impact on the quality of sports. We can see that a strong family sports atmosphere promotes a student’s physical activities. The results of the study are consistent with existing results in that a combined intervention is the most effective, compared with an intervention based solely on school, family, and community [11].

Both intra- and intergroup differences indicate that the intervention had a significant effect on grip strength. The results for the difference between groups showed that the experimental group had 0.519 kg higher right-hand grip strength than the control group. Grip strength is an objective physical function that can be used as a major predictor of health and physical function [40,41] and has become a single marker of physical function that reflects not only arm strength but also whole-body strength. The significant effect for children on grip-strength indicators after 12 weeks of intervention is demonstrated by the fact that the basic characteristics of human movement development were followed in the design of the flipchart content that we developed. The growth and development of children is stage-specific and programmatic—physical fitness is one of the influencing factors—and the sensitive period of physical development determines the logical hierarchy of physical education in children [28]. After the experimental group learned and exercised the contents of the EMWKG flipchart—running, jumping, throwing and catching, climbing, and flexibility—grip strength improved accordingly while promoting the improvement of the muscle level of the whole body. Much of the content in the intervention can be practiced at home, such as the Bobbi jump and parent-child flexibility exercises; these are beneficial to the improvement of strength and flexibility qualities. Particularly for fifth-graders, the EMWKG flipchart contains strength exercises as well as speed, agility, and flexibility exercises, all of which can improve the strength of the whole body. In addition to this, interventions can have a positive effect on students’ attitudes toward exercise, stimulating and increasing their intrinsic motivation to participate in physical activity and exercise, which leads to a certain degree of change in exercise behavior, an increase in exercise time and frequency, and a more scientific and effective approach to exercise. Such a result has been confirmed by studies in which intra-individual indicators such as behavioral habits, behavioral attitudes, and subjective standards are predictors of physical activity and can improve physical activity behavior [35,42].

In the students’ questionnaire responses, the score of the item “Parents often tell me to do exercise” increased. It can be seen that the creation of a good family exercise atmosphere has a positive effect on students’ attitudes and behaviors related to exercise. The study created a parental social support network to indirectly influence students’ exercise attitudes and behaviors through parent–child reading and exercise. It also enabled parents to provide more scientific guidance on their children’s exercise. This study has made some useful attempts to reduce children’s physical inactivity during out-of-school hours and improve children’s motivation to exercise and value physical fitness through parent–child learning and practice together.

In contrast, members of the control group not only did not improve their grip-strength scores but also produced significant decreases in the 50 m run and in 1 min sit-ups. To some extent, it can be shown that the intervention experiment had an enhancing effect on speed as well as on the core strength of the students. We can conclude that the physical exercise and health information leaflet developed for the third- and fifth-grade students in this study not only significantly improved the students’ grip strength but also promoted the students’ speed (50-m dash) and core strength (1-min sit-ups).

Both the experimental and control groups showed significant declines in the seated forward-bend test. The decline may have been influenced by the weather at the time of the test. The correlation between the two has also been confirmed in the study of climate and athletic performance, noting that cold weather can have a detrimental effect on athletic performance [43,44,45]. The first and second tests were conducted in September 2020 and January 2021, respectively, and the performance of the seated forward bend was affected by the students’ wearing thicker clothing and not warming up adequately during the test in the cold of January compared with September when the temperature was warm.

This shows that limitations should be noted. Ignorance of climate effects may have biased the results of the students’ physical fitness tests. The study yielded an unexpected result. As a rule, elementary school students are in a phase of rapid growth, development, and physical improvement, and the expected outcome of the study was that both control and intervention students would improve after one semester, with the intervention group improving more than the control group. However, the experimental results showed that the control group students had significant decreases in physical fitness after one semester. The cold winter months may have had a negative impact on reducing the motivation of students to take physical fitness tests and to practice during the intervention.

An important issue for future consideration is to ensure as much as possible that the environmental conditions are the same for both tests, and it would be good to extend the intervention time to one year. In future research, we will refine the design of the EMWKG flipchart. The flipchart will be designed for each grade level in elementary school and will contain Volume I and Volume II corresponding to the spring and fall semesters, respectively. The follow-up study will extend the intervention duration and validate the effects of the set of EMWKG flipcharts (12 volumes, 2 for each grade level) on students’ attitudes toward exercise and physical fitness through a one-year intervention experiment with groups of students in grades 1–6, respectively.

In addition, future research will explore the impact of the EMWKG flipchart on parents and the mechanisms by which the flipchart influences children through their parents. For example, in addition to observing the changes in parents’ social support perceived by students, parents’ feedback on the intervention will also be included in the variables to explore the changes in parents’ attitudes towards exercise, exercise behavior and physical health after the flipchart intervention.

Accordingly, the results from this study are significant in several respects. The positive effect of the flipchart designed in this study on students’ attitudes toward exercise and physical fitness has been validated. Therefore, the flipchart can be used as a practical tool for the follow-up study intervention. On the other hand, the study provides a theoretical basis for subsequent research on the improvement of students’ physical fitness and exercise attitudes based on a multicomponent social support intervention design and through a home-school approach.

## 5. Conclusions

The strength of this study is that it is a multicomponent intervention based on the four elements of social support theory and targeted at promoting children’s out-of-school exercise behaviors. In this study, the specific intervention combining informational (knowledge in the EMWKG leaflet), instrumental (methods in the EMWKG leaflet and agile ladders), emotional (parental accompaniment), and evolutional (evaluation for the EMWKG leaflet) social support to promote a strong exercise attitude and fitness among children.

The 12-week multicomponent social support intervention significantly improved the behavioral intention and subjective standard subscales of children’s attitudes toward physical activity and improved behavioral cognition, behavioral habits, and affective experiences of physical activity, significantly improving students’ grip strength and promoting both speed quality and core strength. This study has made some useful attempts to reduce children’s physical inactivity during out-of-school hours and improve children’s motivation to exercise and physical fitness through parent–child learning and practice together.

## Figures and Tables

**Table 1:** Design of intervention elements based on social support theory.

Social Support	Contents	Elements of the Intervention
Emotional support		
	The provision of love, trust, care, etc.	Support from parents accompanied by exercise Support from parents accompanied by learning knowledge
Instrumental Support		
	Providing direct and practical help and necessary services	Exercise equipment Exercise methods and guidance Health information booklet Exercise homework
Informational Support		
	Providing consultation, advice, information, etc. on available problems to be solved	Exercise-related knowledge Health-related knowledge
Evaluation Support		
	Feedback, affirmation, and comparative information to facilitate self-evaluation	Learning and post-workout feedback

**Table 2:** Themes and content of the EMWKG leaflets.

Theme	Content
FMS (Fundamental Movement Skill)	Traveling Bending, stretching, curling, and twisting Jumping and landing Balancing Chasing, fleeing, and dodging Throwing and catching
Wellness knowledge	Knowledge of physical activity Exercise and nutrition (proper diet, the role of physical activity in improving the body shape) Exercise and weight Exercise and prevention of disease Exercise and eye health Sports injuries (self-protection) Building good body shape and body posture (understanding height to weight ratio, scoliosis) Sedentary lifestyle and associated hazards
Social adaptation	Overcoming difficulties (endurance running) Unity and cooperation Sports ethics, sportsmanlike behavior (pointing out uncivilized and rule-breaking behaviors) Exercise habits (developing a personal exercise plan) Regulating one’s emotions in sports

**Table 3:** Participant characteristics.

	Intervention Group *n* = 183	Control Group *n* = 183	Total *n* = 366	*p*
Grade				
7	94 (51.4%)	92 (50.3%)	186	0.792
8	89 (48.6%)	91 (49.7%)	180	
Gender				
Boys	98 (53.6%)	94 (51.4%)	183	0.445
Girls	85 (46.4%)	89 (48.6%)	183	
Hight (cm)	139.94 (8.95)	139.84 (9.11)	139.89 (9.02)	0.791
Weight (kg)	33.00 (8.07)	33.23 (8.27)	33.11 (8.16)	0.525
BMI (kg/m^2^)	16.66 (2.69)	16.79 (2.69)	16.73 (2.69)	0.795
Age (year)				
Age, Mean (SD)	9.37 (1.14)	9.33 (1.11)	9.35 (1.12)	0.584
8	60 (33.3%)	60 (32.8%)		
9	31 (16.9%)	32 (17.5%)		
10	60 (32.8%)	54 (29.5%)		
11	31 (16.9%)	37 (20.2%)		

**Table 4:** Phases of the intervention program.

Week	Grade 3	Grade 5
1	Various running exercises	Speed quality exercises
2	Various jumping exercises	Agility exercises
3	Flexibility exercises	Flexibility exercises
4	Throwing exercises	Strength exercises (I)
5	Climbing exercises	Strength exercises (II)
6	Catching exercises	Endurance exercises
7	Exercise and nutrition	Develop a personal fitness program
8	Exercise and weight	Exercise and weight
9	Exercise and disease prevention	Exercise and disease prevention
10	Exercise and eyes health	Exercise and eye health
11	Exercise and safety	Exercise and injury (self-protection)
12	Exercise and social adaptation	Building good body shape and body posture

**Table 5:** Descriptive statistics of the physical fitness variables (mean, standard deviation); within-group analysis.

Variables	Exp			Con		
	Pre	Post	d	Pre	Post	d
Left-hand grip strength (kg)	8.62, 3.34	8.94, 3.39	0.10 *	8.72, 3.18	8.89, 4.09	0.05
Right-hand grip strength (kg)	9.52, 3.55	9.83, 3.61	0.09 *	9.98, 3.31	9.73, 3.89	0.07
50 m (s)	10.14, 1.05	10.14, 1.14	<0.01	9.95, 0.89	10.14, 1.12	0.19 **
Rope-skipping (pcs)	178.23, 20.57	176.93, 21.23	0.06	182.32, 18.42	182.41, 22.02	<0.01
Sit-ups (pcs)	42.02, 11.97	41.24, 10.30	0.07	42.87, 12.15	41.20, 11.32	0.14 *
Sit-and-reach (cm)	16.16, 5.05	14.58, 5.43	0.3 ***	15.60, 5.14	13.06, 5.89	0.46 ***

Note: statistical significance was set to *p* < 0.05; *: *p* < 0.05; **: *p* < 0.01; ***: *p* < 0.001.

**Table 6:** Analysis of covariance results for the physical fitness test indexes between the experimental and control groups.

Variables	Group		Gender		Interaction Effects		*Pairwise* *Comparison*
	*F*	*η* ^2^ *_p_*	*F*	*η* ^2^ *_p_*	*F*	*η* ^2^ *_p_*	*(Post)*
Left-hand grip strength (kg)	0.229	0.001	1.266	0.004	2.082	0.006	——
Right-hand grip strength (kg)	7.109	0.020 ***	2.194	0.006	2.083	0.006	Exp > Con
50 m (s)	1.678	0.005	2.060	0.006	0.024	<0.001	——
Rope-skipping (pcs)	2.997	0.008	15.007	0.041 ***	<0.001	<0.001	——
Sit-ups (pcs)	0.215	0.001	1.932	0.005	1.169	0.003	——
Sit-and-reach (cm)	11.255	0.031 ***	17.720	0.048 ***	3.273	0.009	Exp > Con

Note: The *p* value is the result of the covariance test, setting age, BMI, and baseline test as covariates and group and gender as a fixed factor. ***: *p* < 0.001.

**Table 7:** Descriptive statistics of exercise attitude (mean, standard deviation); within-group analysis.

Variables	Exp			Con		
[Score]	Pre	Post	d	Pre	Post	d
Behavioral attitudes	34.53, 5.27	34.05, 6.34	0.08	35.76, 4.47	34.84, 6.51	0.16
Target attitudes	53.02, 6.60	52.24, 7.89	0.11	54.58, 6.62	52.36, 8.33	0.30 ***
Behavioral perceptions	28.78, 5.81	29.27, 5.75	0.08	30.68, 4.52	29.27, 6.25	0.26 **
Behavioral habits	38.30, 8.66	39.13, 9.50	0.09	39.56, 8.24	38.94, 9.43	0.07
Behavioral intention	29.22, 7.62	30.53, 7.62	0.17 **	30.55, 7.06	30.28, 7.93	0.04
Emotional experience	39.11, 9.30	39.50, 10.17	0.04	40.94, 8.85	39.97, 10.66	0.10
Sense of behavioral control	32.26, 6.50	31.70, 7.22	0.08	33.07, 6.02	31.98, 6.93	0.17 *
Subjective standards	20.71, 5.99	21.71, 6.96	0.15	21.14, 6.23	19.28, 6.34	0.30 ***

Note: Statistical significance was set to *p* < 0.05; *: *p* < 0.05; **: *p* < 0.01; ***: *p* < 0.001.

**Table 8:** Analysis of covariance of the exercise attitude test indexes between the experimental and control groups.

Variables [Score]	Group		Gender		Interaction effects		*Pairwise* *Comparison*
	*F*	*η* ^2^ *_p_*	*F*	*η* ^2^ *_p_*	*F*	*η* ^2^ *_p_*	*Post*
Behavioral attitudes	0.015	<0.001	0.074	<0.001	0.463	0.001	——
Target attitudes	0.977	0.003	0.019	<0.001	1.205	0.004	——
Behavioral perceptions	3.192	0.009	0.013	<0.001	0.382	0.001	——
Behavioral habits	2.400	0.007	0.610	0.002	1.160	0.003	——
Behavioral intention	3.642	0.011	0.859	0.003	0.220	0.001	——
Emotional experience	0.541	0.002	0.007	<0.001	0.900	0.003	——
Sense of behavioral control	0.036	<0.001	0.026	<0.001	0.161	<0.001	——
Subjective standards	15.461	0.043 ***	5.518	0.016 *	0.542	0.002	Exp > Con

Note: The *p* value is the result of the covariance test, setting age, BMI, and baseline test as covariates and group and gender as fixed factors; *: *p* < 0.05; ***: *p* < 0.001.

## Data Availability

The datasets used and/or analyzed during the current study are available from the corresponding author upon reasonable request.
